# Association between weekend catch-up sleep and metabolic syndrome: A cross-sectional study

**DOI:** 10.1097/MD.0000000000049299

**Published:** 2026-06-26

**Authors:** Jingyi Xie, Yixin Guo, Xianyu Xie, Fengjuan Zhou

**Affiliations:** a Zhuhai Campus of Zunyi Medical University, Zhuhai, Guangdong, China; b Division of Birth Cohort Study, Guangzhou Women and Children’s Medical Center, Guangzhou Medical University, Guangzhou, China; c College of Big Data and Computer Science, Guangdong Baiyun University, Guangzhou, China.

**Keywords:** cross-sectional study, metabolic syndrome, NHANES, sleep, weekend catch-up sleep

## Abstract

The association between weekend catch-up sleep (WCS) and the prevalence of metabolic syndrome (MetS) remains controversial. This study aimed to investigate the association between WCS and MetS. This cross-sectional study was performed on individuals (n = 7658) based on the National Health and Nutrition Examination Survey from 2017 to 2020. Five categories of WCS were calculated: decreased (WCS < 0 hours), no change (WCS = 0 hours), short WCS (0 hours < WCS ≤ 1 hour), moderate WCS (1 hour < WCS < 2 hours), and long WCS (WCS ≥ 2 hours). Multivariate logistic regression models were used to evaluate the association between WCS and MetS, as well as its components. The analysis was further refined by integrating a restricted cubic spline, and subgroup and sensitivity analyses were then performed. The study found that moderate (1–<2 hours; odds ratio [OR] = 0.72, 95% confidence interval [CI]: 0.53–0.98) and long (≥2 hours; OR = 0.71, 95% CI: 0.52–0.98) weekly sleep durations were associated with lower odds of MetS. This association appeared to be primarily driven by a robust association with hypertension (moderate WCS: OR = 0.64, 95% CI: 0.46–0.89; long WCS: OR = 0.60, 95% CI: 0.43–0.83). In exploratory subgroup analyses, associations were observed for males, Mexican Americans, individuals who slept 6 to 9 hours/night during the weekday, and those who were sedentary for 8 hours/day; however, interaction tests were not statistically significant for most subgroups, and these findings require further validation. Sensitivity analyses confirmed the stability of the association between moderate/long WCS and lower odds of MetS. The restricted cubic spline model indicated a nonlinear relationship between WCS and MetS. Moderate to long WCS was associated with lower odds of hypertension and MetS in this cross-sectional analysis. Additional prospective studies are required to verify these findings and elucidate the underlying mechanisms.

## 1. Introduction

Metabolic syndrome (MetS) includes a combination of clustered abnormalities, usually composed of central obesity, high blood glucose, high blood pressure, hypertriglyceridemia, and low high-density lipoprotein cholesterol (HDL-C) levels.^[1]^ MetS has become a global health concern. A recent study revealed that MetS affects over 1 billion individuals globally, including 35% of adult Americans. MetS is associated with adverse health consequences. Research indicates that individuals with MetS have elevated cardiovascular/cerebrovascular disease risks and triple to sextuple mortality rates.^[2]^ Moreover, the significant impact of MetS on healthcare consumption has been recorded, with healthcare costs reported to rise as much as 60% for patients.^[3,4]^

Sleep plays a critical role in the development of MetS. An adequate duration is essential for metabolic homeostasis, cognitive performance, and longevity.^[5,6]^ In contrast, both sleep deprivation (<6 hours) and hypersomnia (>9 hours) have been linked to MetS.^[7]^ Currently, an interesting phenomenon that has become known during weekdays is weekend catch-up sleep (WCS), or compensatory catch-up sleep on weekends, reported by those living in modern lifestyles. This phenomenon concerns sleeping longer on weekends to compensate for sleep loss during the workweek. WCS means that people extend their sleeping time on the weekend to catch up with the sleep duration on weekdays.

Emerging evidence indicates that moderate WCS may assist in counteracting metabolic disturbances caused by weekday deficits, whereas excessive WCS has the potential to disrupt circadian rhythms, compromise sleep quality, and exacerbate inflammation or hormonal imbalance.^[8,9]^ The findings of these studies suggest that moderate WCS is associated with favorable metabolic outcomes. The combined findings of 2 studies, 1 conducted by Kim et al^[10]^ and the other by Luo et al^[11]^ revealed that daily WCS exceeding 1 hour correlates with a lower hypertension risk. Those studies found that moderate WCS (1–2 hours) was associated with better glycemic control and a lower risk of prediabetes, whereas excessive WCS (≥3 hours) was associated with impaired glucose regulation and a higher risk of diabetes.^[12]^ Similarly, among individuals with less than 6 hours of weekday sleep, WCS exceeding 2 hours has been linked to a reduced prevalence of cardiovascular disease (CVD).^[13]^ Previous research in South Korea indicated a relationship between WCS and the various components of MetS. A study utilizing data from South Korea identified a correlation between WCS and a reduced MetS prevalence in adults experiencing insufficient weekday sleep (<9 hours), although this association was not observed in those with adequate or prolonged sleep (≥9 hours). The optimal sleep pattern for the prevention of MetS has been identified as 6 to 7 hours of sleep on weekdays combined with ≥2 hours of WCS.^[14]^ Another study focusing on individuals with weekday sleep duration of less than 6 hours suggested that WCS of at least 40 minutes may be associated with lower odds of MetS, particularly in populations.^[9]^

However, many critical limitations have fragmented the existing evidence, and most studies have focused on South Korean populations, which differ from the United States in terms of lifestyle patterns and demographic diversity. Furthermore, most previous studies have inadequately addressed the classification complexity of MetS across multi-ethnic populations. Using National Health and Nutrition Examination Survey (NHANES) 2017 to 2020 data, we examined the relationship between WCS and MetS, guided by 2 a priori hypotheses: (H1) a nonlinear dose-response association exists between WCS duration and MetS and (H2) demographic and lifestyle factors modify the association between moderate WCS and MetS.

## 2. Methods

### 2.1. Study participants

Data for this research were drawn from the NHANES 2017 to March 2020 pre-pandemic data, an extensive multistage sample survey implemented by the National Center for Health Statistics to assess the nutrition and health status of adults and children living in the United States. The portions of this study involving human participants, materials, or data were conducted in accordance with the Declaration of Helsinki and were approved by the National Center for Health Statistics Ethics Review Board (protocol numbers: Protocol #2011–17, Protocol #2018–01). Each participant provided informed consent before participating in the study. Detailed instructions can be found on the official website at https://www.cdc.gov/nchs/nhanes/.^[15,16]^

**Figure 1.**
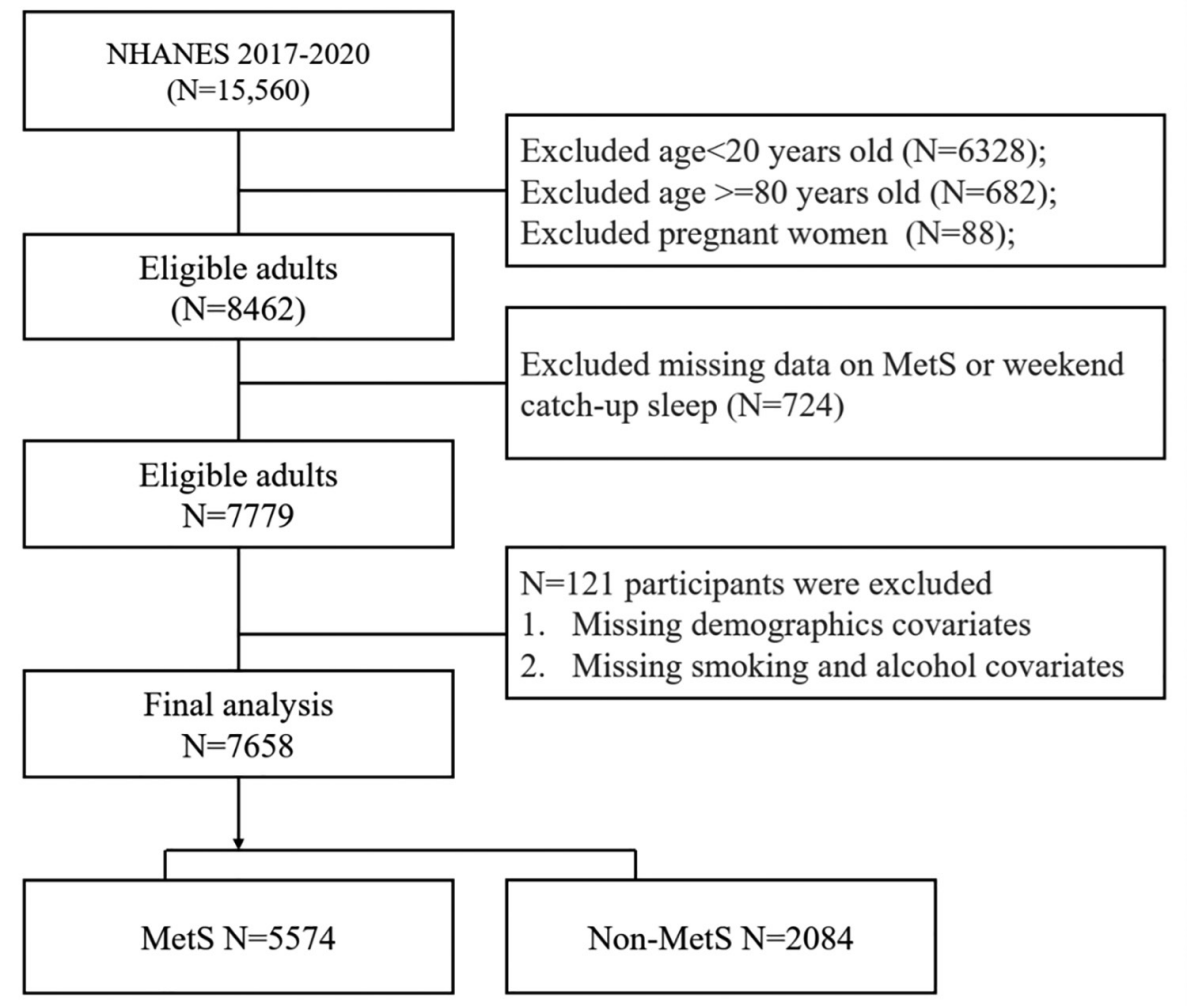
Flowchart of participant selection from the 2017 to 2020 NHANES dataset. The chart illustrates the process of applying the inclusion and exclusion criteria, resulting in a final sample of 7658 participants for the analysis. MetS = metabolic syndrome, NHANES = National Health and Nutrition Examination Survey.

We selected and analyzed the data according to specific criteria. Inclusion criteria: Participants were required to have complete data on WCS and MetS in their questionnaires. The exclusion criteria were as follows: age <20 or ≥80 years, pregnancy, missing data on MetS or WCS in the questionnaire, and missing data on the covariates (demographics, smoking history, and alcohol consumption). Applying the above criteria yielded 7658 participants in this study. The detailed process is illustrated in Figure 1.

### 2.2. WCS

WCS represented the arithmetic differential between weekend and weekday sleep periods in the NHANES 2017 to 2020 dataset via self-reported measures provided in the “Sleep Disorders Questionnaire.” The measurement process was conducted in 3 steps: Sleep duration assessment: The average number of hours spent sleeping each weekday and weekend was recorded. WCS calculation: WCS was calculated as the difference between self-reported average weekend sleep duration and average weekday sleep duration. A positive WCS value indicated longer sleep duration on weekends than on weekdays, reflecting WCS behavior.

We identified 5 categories for WCS scoring: decreased (WCS < 0 hours), no change (WCS = 0 hours), short catch-up (0 hours < WCS ≤ 1 hour), moderate catch-up (1 hour < WCS < 2 hours), and long catch-up (WCS ≥ 2 hours).^[17]^

### 2.3. MetS

MetS identification was classified according to NCEP ATP III specifications.^[1]^ NHANES data were used with established dimensional criteria to assess waist circumference (WC), high blood pressure, fasting blood glucose, and lipid profiles. MetS was determined when 3 or more of the following conditions existed simultaneously: central obesity defined by WC > 102 centimeters for males and >88 centimeters for females; glucose dysmetabolism as shown by history of type 2 diabetes or fasting plasma glucose > 5.6 mmol/L; hypertension specified as having systolic/diastolic proportions >130/85 mm Hg or taking antihypertensive medications; high triglycerides (TGs) ≥1.70 mmol/L while fasting; and low HDL-C: <1.03 mmol/L in males or <1.30 mmol/L in females.

### 2.4. Covariates

In this analysis, the variables were readjusted and considered to manage potential confounding factors. The covariates included: demographic variables: age groups (20–44, 45–64, or ≥65 years), sex, race (non-Hispanic Blacks, non-Hispanic Whites, Mexican Americans, and other races), marital status (“Married or living with partner” and “Marital disruption [widowed/divorced/separated]”); socioeconomic variables: educational attainment (below high school vs high school or above); lifestyle variables: smoking status was classified as never, former, or current smoker. Alcohol consumption was categorized based on self-reported drinking frequency and quantity into light, moderate, or heavy drinkers^[18]^; obstructive sleep apnea (OSA),^[19,20]^ yes or no; social jetlag (calculated as the absolute difference between the midpoint of sleep on workdays and weekend days, categorized as <2 hours or ≥2 hours); and sleep duration: weekday sleep duration was divided into 3 groups: short (≤6 hours), adequate (>6 but <9 hours), and long sleepers (≥9 hours).^[21]^ Specific definitions of these covariates are provided in Table S1, Supplemental Digital Content 1.

### 2.5. Statistical analysis

Descriptive statistics were expressed as weighted proportions for categorical variables and weighted means with standard errors for continuous variables. This study employed the “WTMECPRP” variable from the 2017 to 2020 NHANES cycle as the sampling weight to ensure representativeness. Chi-squared tests were applied to the survey data to detect differences between groups with and without WCS.

The primary analytical approach involved weighted logistic regression to examine the odds ratios (ORs) of different WCS durations for MetS and its main components, with a decreased WCS (< 0 hours) as the reference group. Three sequential models were constructed: Model 1: an unadjusted (crude) model; Model 2: adjusted for demographic factors (sex, age, and race); and Model 3: Model 2 + socioeconomic/behavioral covariates (education, marital status, smoking, alcohol use, sedentary behavior, OSA, and social jetlag).

To explore the potential nonlinear associations between WCS and MetS, as well as its components, we used restricted cubic splines (RCSs) with weighted generalized additive models. Marginal ORs and 95% confidence intervals (CIs) were calculated using the “emmeans” package, which provides population-averaged effect estimates averaged over the distribution of all covariates in the model. This provides a single, population-averaged estimate of the effect for each WCS category. Stratified analyses were performed based on sex, age groups, race, weekday sleep duration, sedentary behavior categories, smoking, alcohol categories, and OSA status. In addition, we performed sensitivity analyses for people who slept between 3 and 12 hours during the week.^[22,23]^ All statistical analyses were conducted using *R*-4.2.2 (R Core Team).

## 3. Results

### 3.1. Characteristics of study participants

Sociodemographic information and characteristics are presented in Table 1. The analysis included 2084 MetS cases and 5574 non-MetS controls. The participants were 47 (standard deviation 0.5) years old, with 51.08% female. A total of 80% of the sample were married or living with a partner, and 96.5% were educated to high school or above. The mean (standard deviation) duration was 0.69 (0.03). People with MetS were older, had less schooling, were fatter, and spent more time sitting. In addition, participants with MetS had higher body mass index, WC, TG, fasting glucose, and blood pressure, and lower HDL-C levels. No significant difference in the total WCS duration was observed between the 2 groups (*P* = .75). However, there was a trend toward a reduced prevalence of MetS in the long WCS (≥2 hours) group (17.54% vs 22.80%, *P* = .01).

**Table 1:** Characteristics of participants in the NHANES 2017 to 2020 cycles (n = 7658).

Variable	Total (7658)	Non-MetS (5574)	MetS (2084)	*P* value
Age, yr	47.00 ± 0.50	44.52 ± 0.49	54.74 ± 0.65	<.001
Age group, n (%)				<.0001
20–44 yr	3118 (45.71)	2681 (52.44)	437 (24.77)	
45–64 yr	3002 (36.92)	1998 (33.81)	1004 (46.63)	
≥65 yr	1538 (17.37)	895 (13.76)	643 (28.60)	
Sex, n (%)				.22
Female	3926 (51.08)	2770 (50.40)	1156 (53.23)	
Male	3732 (48.92)	2804 (49.60)	928 (46.77)	
Race, n (%)				.46
Mexican American	934 (8.52)	654 (8.48)	280 (8.62)	
Non-Hispanic Black	2094 (11.35)	1517 (11.49)	577 (10.91)	
Non-Hispanic White	2458 (62.26)	1765 (61.74)	693 (63.91)	
Other	2172 (17.87)	1638 (18.29)	534 (16.56)	
Education, n (%)				<.001
Below high school	566 (3.47)	355 (3.01)	211 (4.90)	
High school education or more	7087 (96.50)	5216 (96.99)	1871 (95.10)	
Living with partner, n (%)				<.001
Yes	6086 (80.06)	4293 (77.54)	1793 (87.91)	
No	1572 (19.94)	1281 (22.46)	291 (12.09)	
BMI group, n (%)				<.0001
Normal	1927 (26.41)	1833 (33.65)	94 (3.84)	
Overweight	2410 (31.37)	1885 (33.56)	525 (24.56)	
Obesity	3321 (42.22)	1856 (32.79)	1465 (71.60)	
Weekday sleep duration	7.53 (0.03)	7.53 (0.03)	7.53 (0.05)	.93
Weekday sleep duration, n (%)				.13
6–9 h	4489 (64.22)	3328 (65.27)	1161 (60.96)	
≤6 h	1542 (17.18)	1092 (16.56)	450 (19.10)	
≥9 h	1627 (18.60)	1154 (18.17)	473 (19.94)	
Weekend sleep duration	8.22 (0.03)	8.23 (0.04)	8.20 (0.06)	.75
WCS	0.69 (0.03)	0.70 (0.03)	0.67 (0.03)	.55
WCS, n (%)				.01
Decreased	1714 (20.95)	1272 (20.90)	442 (21.10)	
No change	1234 (15.12)	911 (14.97)	323 (15.58)	
Short	436 (6.51)	331 (6.55)	105 (6.36)	
Moderate	2785 (35.90)	1933 (34.78)	852 (39.41)	
Long	1489 (21.52)	1127 (22.80)	362 (17.54)	
Social jetlag, n (%)				.002
No	5965 (79.27)	4259 (78.41)	1706 (83.85)	
Yes	1620 (20.15)	1263 (21.59)	357 (16.15)	
OSA, n (%)				<.0001
No	3789 (50.65)	2986 (54.65)	803 (38.18)	
Yes	3869 (49.35)	2588 (45.35)	1281 (61.82)	
Smoke, n (%)				.005
Former	1704 (25.06)	1122 (22.81)	582 (32.07)	
Never	4499 (57.59)	3369 (59.83)	1130 (50.59)	
Now	1455 (17.35)	1083 (17.36)	372 (17.33)	
Alcohol, n (%)				<.0001
Mild	1275 (18.53)	977 (19.67)	298 (14.95)	
Moderate	4727 (55.36)	3310 (52.29)	1417 (64.91)	
Heavy	1656 (26.12)	1287 (28.04)	369 (20.14)	
Sedentary behavior, n (%)				<.0001
<4 h	2481 (27.62)	1858 (29.06)	623 (23.71)	
4–8 h	3805 (51.18)	2751 (50.88)	1054 (53.20)	
≥8 h	1325 (20.69)	933 (20.06)	392 (23.09)	
WC, cm	100.52 ± 0.52	96.42 ± 0.49	113.07 ± 0.67	<.0001
SBP, mm Hg	121.33 ± 0.36	119.52 ± 0.34	126.76 ± 0.57	<.0001
DBP, mm Hg	74.21 ± 0.27	73.19 ± 0.31	77.31 ± 0.32	<.0001
FG, mmol/L	6.06 ± 0.05	5.60 ± 0.02	6.91 ± 0.10	<.0001
TGs, mmol/L	1.25 (0.03)	0.96 (0.02)	1.80 (0.06)	<.0001
HDL-C, mmol/L	1.38 ± 0.01	1.46 ± 0.01	1.15 ± 0.01	<.0001

BMI = body mass index, DBP = diastolic blood pressure, FG = fasting glucose, HDL-C = high-density lipoprotein cholesterol, MetS = metabolic syndrome, NHANES = National Health and Nutrition Examination Survey, OSA = obstructive sleep apnea, SBP = systolic blood pressure, TGs = triglycerides, WC = waist circumference, WCS = weekend catch-up sleep.

Baseline characteristics stratified by the WCS groups are detailed in Table S2, Supplemental Digital Content 2. These groups differed in age, racial distribution, and most notably, social jetlag, where the “No-change” group had the highest proportion of social jetlag (53.30%).

**Figure 2.**
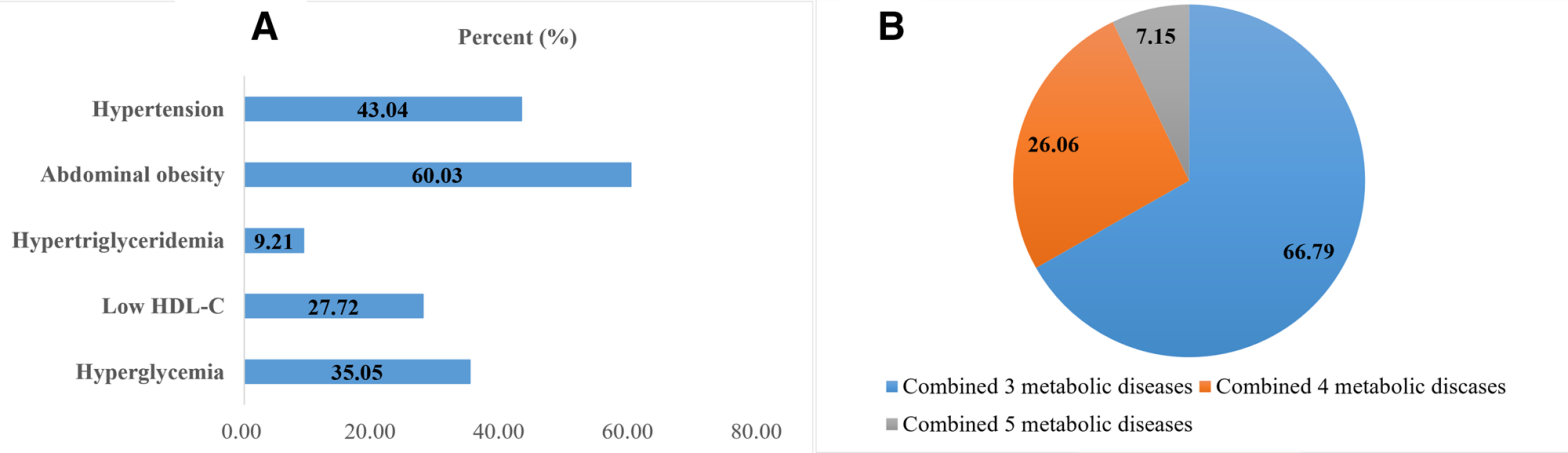
Weighted prevalence of metabolic syndrome (MetS) components. (A) Weighted percentage of individual MetS components among the study population. Abdominal obesity was the most prevalent. (B) Distribution of the number of concurrent metabolic abnormalities among patients with MetS. HDL-C = high-density lipoprotein cholesterol.

The prevalence of MetS and its diagnostic components is shown in Figure 2. Abdominal obesity was the most prevalent condition (57.55%), followed by hypertriglyceridemia (9.73%; Fig. 2A). A total of 66.79% of the patients with MetS exhibited 3 concurrent metabolic abnormalities, 26.06% had 4 components, and 7.15% had all 5 components (Fig. 2B).

### 3.2. Associations between WCS and MetS and its components

The univariate analysis revealed a significant association between MetS and several covariates. Age was strongly associated, with older age groups (45–64 and ≥65 years) having higher odds. Educational level and marital status (living with partners), OSA, and social jetlag also showed significant associations. Sedentary behavior and smoking status were also associated with MetS. However, sex, race, drinking, and sleeping duration were not associated (see Table S3, Supplemental Digital Content 3).

**Figure 3.**
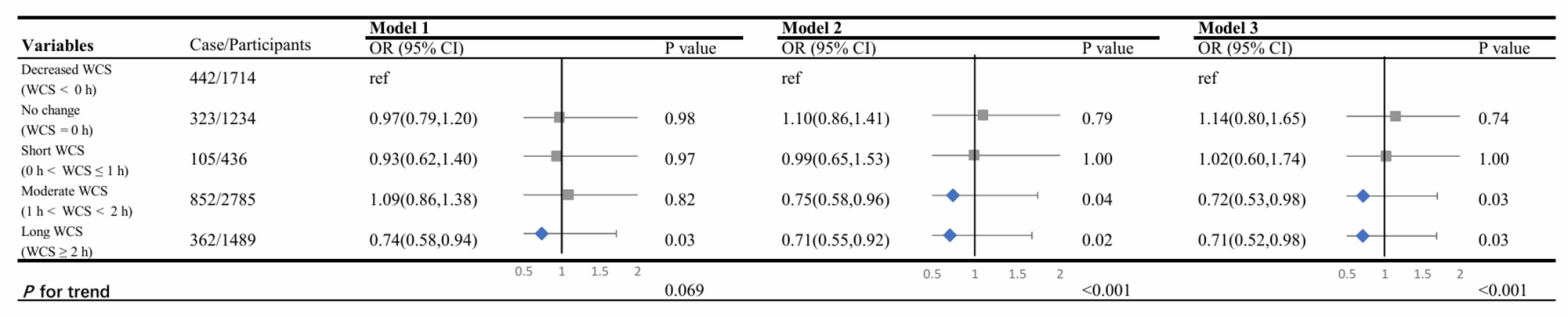
Association between WCS categories and MetS. Forest plot showing the marginal ORs with 95% CIs. Results are shown for unadjusted (Model 1), partially adjusted (Model 2), and fully adjusted (Model 3) analyses. The “Decreased WCS (<0 hours)” category serves as the reference. CI = confidence interval, MetS = metabolic syndrome, OR = odds ratio, WCS = weekend catch-up sleep.

As shown in Figure 3, multivariable-adjusted analyses revealed significant associations between WCS and MetS. In unadjusted Model 1, no change (WCS = 0 hours) and short and moderate WCS were not associated with MetS. Long WCS (≥2 hours) was associated with MetS (OR 0.74). After full adjustments in Model 3, the results showed that moderate and long WCS were associated with MetS (ORs 0.72 and 0.71), while decreased and short WCS did not show significant associations (ORs 1.14 and 1.02, respectively).

Multivariable-adjusted analyses of individual components of MetS are shown in Table S4, Supplemental Digital Content 4. After fully adjusting for covariates in Model 3, the results showed that moderate and long WCS were associated with a lower likelihood of hypertension (ORs = 0.64 and 0.60, respectively).

### 3.3. Restricted cubic splines

**Figure 4.**
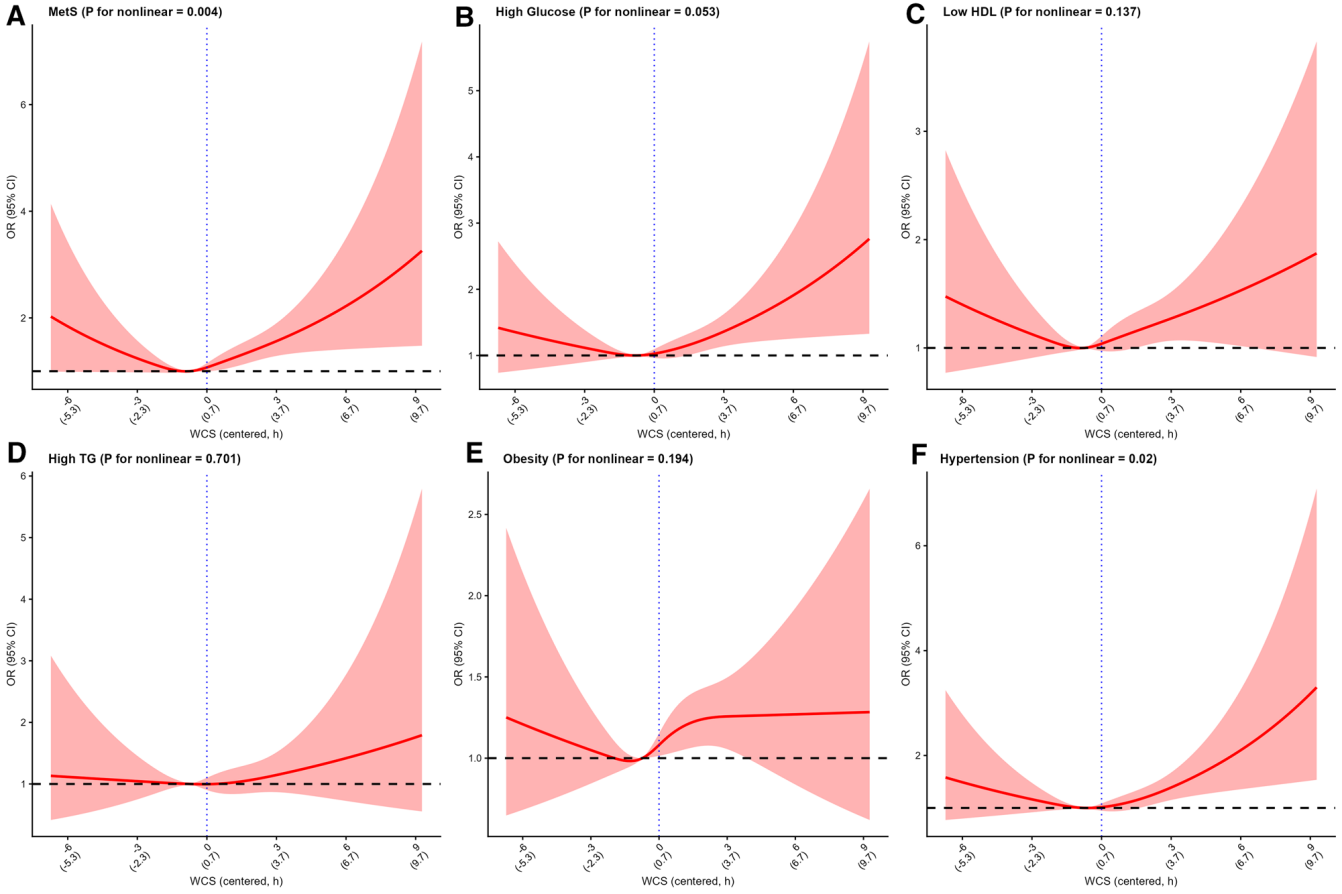
Restricted cubic spline (RCS) analysis of the association between WCS duration and MetS and its components. The analysis shows the adjusted odds ratio (solid red line) and 95% confidence interval (shaded area) for MetS and its components across the continuous spectrum of WCS. The x-axis represents the mean-centered WCS time in hours. The horizontal dashed line indicates an OR of 1. (A) MetS; (B) hyperglycemia; (C) low HDL-C; (D) high TG; (E) abdominal obesity; (F) hypertension. CI = confidence interval, HDL-C = high-density lipoprotein cholesterol, MetS = metabolic syndrome, OR = odds ratio, TG = triglycerides, WCS = weekend catch-up sleep.

Using RCS regression, we examined the nonlinear associations between mean-centered WCS and MetS components. The x-axis represents the mean-centered WCS (hours), where 0 corresponds to the mean WCS duration in our study; negative values indicate shorter WCS than the mean, and positive values indicate longer WCS than the mean. The analysis revealed a significant nonlinear relationship with MetS (*P* for nonlinearity = .004), where the odds decreased progressively as WCS increased up to approximately 2 hours, after which the curve plateaued (Fig. 4A). As depicted in Figure 4F, a similar significant nonlinear pattern was observed for hypertension risk (*P* for nonlinearity = .02). For other MetS components, including high glucose (*P* for nonlinearity = .053; Fig. 4B), low HDL (*P* for nonlinearity = .137; Fig. 4C), high TGs (*P* for nonlinearity = .701; Fig. 4D), and obesity (*P* for nonlinearity = .194; Fig. 4E), no statistically significant nonlinearity was detected. Detailed results for all MetS components are available in Table S4, Supplemental Digital Content 4.

It is important to explicitly distinguish between the comparative frameworks for categorical and continuous analyses. The categorical analysis (Fig. 3) compares each WCS category to the decreased WCS group (WCS < 0), demonstrating an association between catching up on sleep and MetS. In contrast, the RCS analysis (Fig. 4) models the risk across the continuous spectrum of WCS, with the population mean WCS set as the reference (OR = 1 at the mean). This indicates that deviations from the mean WCS in either direction are associated with higher odds of MetS, a finding that complements the results of the categorical analysis.

### 3.4. Subgroup analysis

All subgroup analyses were prespecified as exploratory, given that only the interaction test for race/ethnicity reached statistical significance (*P* for interaction = .006), while interaction tests for all other stratification variables were nonsignificant (all *P* for interaction > .05). Stratified analyses adjusted for all covariates in Model 3 were conducted across subgroups defined by age, sex, race/ethnicity, weekday sleep duration, sedentary time, alcohol consumption, social jetlag, and OSA status (full results in Table S5, Supplemental Digital Content 5).

For the subgroup with 6 to 9 hours of sleep during weekdays, both moderate WCS (OR = 0.60) and long WCS (OR = 0.63) were associated with lower odds of MetS. In male participants, moderate WCS was associated with lower odds of MetS (OR = 0.62). For the Mexican American subgroup, long WCS was associated with lower odds of MetS (OR = 0.59). Descriptive data on social jetlag across racial groups are shown in Table S6, Supplemental Digital Content 6. When participants spent ≥8 hours/day engaging in sedentary behavior, long WCS was associated with lower odds of MetS (OR = 0.38). Additionally, long WCS was associated with lower odds of MetS for participants with mild alcohol use (OR = 0.34). In participants without OSA, both moderate (OR = 0.66, *P* = .06) and long WCS (OR = 0.62, *P* = .05) were associated with lower odds of MetS, though with borderline statistical significance. Among individuals with social jetlag, moderate WCS was associated with lower odds of MetS (OR = 0.34, *P* = .01).

### 3.5. Sensitivity analysis

The details of the sensitivity analysis investigating the relationship between weekday sleep duration within a strict range (3–12 hours) and MetS components, categorized by WCS, are shown in Table S7, Supplemental Digital Content 7. Sensitivity analysis confirmed that the associations between moderate/long WCS and lower odds of MetS and hypertension remained robust.

## 4. Discussion

Utilizing nationally representative data from NHANES 2017 to 2020, this study provided unique insights into the associations between WCS and MetS across various population subgroups. The analysis demonstrated associations between moderate and long WCS durations and MetS and hypertension. These findings remained consistent even after adjusting for potential confounders, underscoring that WCS may be a modifiable factor associated with metabolic health. Compared with participants who had reduced weekend sleep (WCS < 0 hours), those with moderate or long WCS showed lower odds of MetS. Conversely, the RCS analysis employed mean-centered WCS, designating the population average as the reference point (OR = 1). This continuous model showed that the more obvious the deviation from the average WCS of the population, whether shorter or longer, the higher the probability of MetS occurrence. This “U-shaped” pattern reflects a deviation from the population mean and should not be misinterpreted as a direct contrast to the fixed pattern of consistent sleep (WCS = 0).

The conclusions of the present study align with those of most previous studies. For instance, a study focusing on individuals with a short sleep duration (<6 hours) found that WCS was associated with lower odds of MetS. The present study focused on individuals aged 35 to 60 years, and these findings were consistent with those of another study that primarily targeted individuals aged 20 to 65 years.^[9,24]^ The aforementioned studies exclusively focused on individuals with short sleep durations and utilized WCS as a categorical variable. In contrast, our study encompassed a broader age range, from 20 to 80 years, and incorporated both continuous and categorical variables for WCS. However, a study from the Korea National Health and Nutrition Examination Survey involving 24,313 participants reported findings not entirely consistent with our study.^[14]^ The association between WCS and MetS odds exhibited significant heterogeneity across weekday sleep duration strata. In individuals reporting 6 to 9 hours of weekday sleep, moderate and long WCS were associated with lower MetS odds compared with no change; however, no consistent patterns were observed among those sleeping ≤6 or ≥9 hours. The divergent findings between this study and the KNHANES could stem from our smaller sample size, as well as the more racially homogeneous population in the KNHANES. Moreover, the percentage of people with ≤6 hours of sleep in the KNHANES population exceeded 50%, whereas in our study, this proportion was just 17%.

MetS is characterized by the co-occurrence of central obesity, hyperglycemia, hypertension, and dyslipidemia. It has been suggested that these elements dramatically increase the incidence of heart disease, stroke, and other public health-related illnesses.^[25]^ In our study, a WCS ≥ 1 hour was associated with hypertension, which is a major factor contributing to the observed association with MetS. Sleep insufficiency has been associated with increased release of inflammatory cytokines, such as tumor necrosis factor-alpha and interleukin-6, which are related to hypertension.^[26]^ Additionally, irregular sleep patterns have been linked to disruption of the balance between the sympathetic and parasympathetic nervous systems, which may contribute to higher blood pressure levels.^[27]^

Therefore, the association between WCS and hypertension may be partly explained by reduced systemic inflammation and restored autonomic nervous system balance, which could in turn relate to lower MetS risk. While WCS may confer short-term metabolic benefits, the potential long-term adverse effects of irregular sleep patterns must be considered when evaluating its overall impact on health. Studies have indicated that weekend sleep recovery is associated with moderate circadian rhythm shifts,^[28]^ which may contribute to misalignment and weekday “social jetlag” upon routine resumption.

A notable observation was the change in the OR for moderate WCS from 1.09 in the unadjusted model to 0.75 after demographic adjustment (Model 2), suggesting confounding by basic demographic factors. Although mean ages were similar across groups, the moderate WCS group had a higher proportion of adults aged ≥65 years (31.93% vs 14.08%), a subgroup with a higher baseline prevalence of MetS.

In subgroup analyses, both moderate and long WCS were associated with lower odds of MetS among Mexican American participants. While this subgroup had the highest prevalence of MetS, it also showed the strongest association with WCS. These exploratory findings may reflect differences in sociocultural factors, health behaviors, or sleep patterns, though further research is needed to clarify the underlying mechanisms.

In exploratory subgroup analyses, an association between moderate WCS and MetS was observed specifically in males, although the test for interaction by sex was not significant. These findings should be interpreted with caution and warrant further investigation. Previous studies have suggested that sleep recovery may influence sympathetic nervous system activity and inflammatory markers and that testosterone levels, which can be affected by sleep duration, are associated with systolic blood pressure in males.^[29]^ In the context of sleep deprivation among males during weekdays, an increase in sympathetic nervous system activity and inflammatory levels has been documented.^[30]^ WCS may contribute to stabilizing the sympathetic nervous system and reducing inflammatory markers, thereby potentially improving metabolic parameters. The observed association in males may be partly attributable to the elevated work and life pressures they commonly experience, which can lead to sleep deprivation, making catch-up sleep potentially more impactful.^[13]^

In exploratory subgroup analyses, the same sort of association was observed for Mexican Americans, who showed an association despite the lowest absolute WCS (0.59 ± 0.03 hours). One possible explanation is that they have more regular sleep patterns, as reflected by the lowest social jetlag prevalence (Table S7, Supplemental Digital Content 7). Additionally, 38.80% of this group achieved moderate catch-up sleep, which may suggest that consistent, small compensation is better than irregular, large catch-up sleep. Other potential contributing factors could include socioeconomic status and culturally specific practices such as family-oriented routines and earlier bedtimes. However, these hypotheses require further investigation, ideally with larger, multi-ethnic cohorts. Therefore, an investigation into ethnic-specific mechanisms is warranted. Additionally, WCS has been observed to be associated with metabolic benefits among individuals engaging in more than 8 hours of daily sedentary behavior. Subsequent investigations should examine the interplay between all daily activities within 24 hours. WCS is part of a broader strategy for optimizing these behaviors to achieve better health outcomes.^[31]^

One of the mechanisms that could contribute to the association between WCS and metabolic outcomes is the hypothalamic-pituitary-adrenal (HPA) axis. Disrupted cortisol release is typically observed in sleep-deprived individuals. It can trigger various unwanted metabolic effects, including abdominal fat accumulation, increased blood pressure, hyperglycemia, and increased TG levels. These effects are associated with HPA axis dysregulation and systemic inflammation.^[32]^ WCS may be associated with recovering HPA axis function and counteracting the unwanted effects of sleep deprivation on metabolic homeostasis and inflammatory responses.^[33,34]^

This study has several limitations. First, as it is a cross-sectional study, it can only show the possibility of an association and not a causal relationship. However, these results provide an opportunity for further longitudinal research on the causality and temporal relationships between WCS and MetS. Second, the assessment of sleep time relied on self-administered questionnaires, which have the potential to lead to recall bias.^[35]^ Third, nap duration was not accounted for in this study. Previous research has demonstrated a U-shaped association between nap duration and MetS, with both short and long naps associated with increased risk.^[36]^ Finally, the cross-sectional design necessitates consideration of reverse causation. MetS and its components (obesity, hypertension, and diabetes) may directly disrupt sleep architecture via sleep apnea,^[36]^ nocturia, medication side effects, and daytime sleepiness, thereby altering WCS patterns. This suggests that MetS may precede WCS behaviors, and longitudinal studies are required to clarify their temporal relationship.

Despite these constraints, the findings of this analysis are still generalizable to the American population, considering our utilization of representative US population data. This study has the potential to add to the current knowledge about the relationship between sleep duration and MetS by showing that WCS may be helpful for MetS based on weekday sleep duration. Longitudinal studies are warranted to further elucidate the relationship between weekday sleep duration, WCS, and MetS risk.

### 4.1. Strengths and limitations of this study

- This study used a large nationally representative sample from the NHANES.
- Cross-sectional design only shows associations, not causality; it paves the way for longitudinal studies on WCS-MetS links.
- Self-reported sleep time risks recall bias.
- Uncontrolled nap duration.

## 5. Conclusion

The present analysis identified associations between WCS and MetS, with the most pronounced relationship observed for hypertension. In exploratory subgroup analyses, numerically stronger associations were observed in individuals with 6 to 9 hours of weekday sleep, males, Mexican Americans, and those with high sedentary behavior, although the interaction tests were not statistically significant. This study provides epidemiological evidence linking regular sleep habits and WCS with metabolic health outcomes. Future studies should establish a timeline and examine physiology, such as circadian rhythms and inflammation.

## Acknowledgments

We are grateful to the founders and participants of the NHANES program.

## Author contributions

**Conceptualization:** Fengjuan Zhou.

**Data curation:** Jingyi Xie, Yixin Guo.

**Methodology:** Yixin Guo.

**Validation:** Yixin Guo, Xianyu Xie.

**Formal analysis:** Fengjuan Zhou.

**Funding acquisition:** Fengjuan Zhou.

**Project administration:** Fengjuan Zhou.

**Writing – original draft:** Jingyi Xie.

**Writing – review & editing:** Jingyi Xie, Yixin Guo, Xianyu Xie, Fengjuan Zhou.














