# Supplementary material for: Association between weekend catch-up sleep and metabolic syndrome: A cross-sectional study
Source: Medicine (Baltimore). 2026 Jun 26;105(26):e49299. doi: 10.1097/MD.0000000000049299 (PMC13313639; doi:10.1097/MD.0000000000049299)
Supplement: Supplementary file 1 [file medi-105-e49299-s001.doc]

**Table S1: Specific definitions and variables in the paper**

| **Section** | **Variable in Paper** | **Variable Type** | **Component** | **NHANES Dataset** | **Items / Description** | **NHANES Variable** |
| --- | --- | --- | --- | --- | --- | --- |
| Demographic | Age group | Categorical | DEMO | DEMO | Age in years at screening (20-44, 45-64, 65+ years) | RIDAGEYR |
| Demographic | Age (years) | Continuous | DEMO | DEMO | Age in years at screening | RIDAGEYR |
| Demographic | Sex | Categorical | DEMO | DEMO | Gender (Male/Female) | RIAGENDR |
| Demographic | Race | Categorical | DEMO | DEMO | Race/Hispanic origin | RIDRETH3 |
| Demographic | PIR | Continuous | DEMO | DEMO | Ratio of family income to poverty | INDFMPIR |
| Demographic | Marital status | Categorical | DEMO | DEMO | Marital status (yes/no) | DMDMARTL |
| Demographic | Educational attainment | Categorical | DEMO | DEMO | Education level - Adults 20+ | DMDEDUC2 |
| Lifestyle | Alcohol intake | Categorical | ALQ | Questionnaire | Ever had a drink of any kind of alcohol | ALQ111 |
| Lifestyle | Alcohol intake | Categorical | ALQ | Questionnaire | How often have you drunk alcohol in the past 12 months? | ALQ121 |
| Lifestyle | Alcohol intake | Categorical | ALQ | Questionnaire | Average number of alcohol drinks per day in the past 12 months | ALQ130 |
| Lifestyle | Alcohol intake | Categorical | ALQ | Questionnaire | Number of days with 4 or 5 drinks in the past 12 months | ALQ142 |
| Lifestyle | Alcohol intake | Categorical | ALQ | Questionnaire | Number of times having 4-5 drinks in 2 hours in the past 12 months | ALQ270 |
| Lifestyle | Alcohol intake | Categorical | ALQ | Questionnaire | # times 8+ drinks in 1 day/past 12 mos | ALQ280 |
| Lifestyle | Alcohol intake | Categorical | ALQ | Questionnaire | # times 12+ drinks in 1 day/past 12 mos | ALQ290 |
| Lifestyle | Alcohol intake | Categorical | ALQ | Questionnaire | Ever have 4/5 or more drinks every day? | ALQ151 |
| Lifestyle | Smoking status | Categorical | SMQ | Questionnaire | Smoked at least 100 cigarettes in life | SMQ020 |
| Lifestyle | Smoking status | Categorical | SMQ | Questionnaire | Do you currently smoke cigarettes? | SMQ040 |
| Lifestyle | Smoking status | Categorical | SMQ | Questionnaire | How long since you quit smoking cigarettes? | SMQ050Q |
| Sleep | WCS | Categorical | SLQ | Questionnaire | Sleep hours - weekdays or workdays | SLD012 |
| Sleep | WCS | Categorical | SLQ | Questionnaire | Sleep hours - weekends | SLD013 |
| Sleep | Social jetlag | Categorical | SLQ | Questionnaire | Usual sleep time on weekdays or workdays | SLQ300 |
| Sleep | Social jetlag | Categorical | SLQ | Questionnaire | Usual wake time on weekdays or workdays | SLQ310 |
| Sleep | Social jetlag | Categorical | SLQ | Questionnaire | Sleep hours - weekdays or workdays | SLD012 |
| Sleep | Social jetlag | Categorical | SLQ | Questionnaire | Usual sleep time on weekends | SLQ320 |
| Sleep | Social jetlag | Categorical | SLQ | Questionnaire | Usual wake time on weekends | SLQ330 |
| Sleep | Social jetlag | Categorical | SLQ | Questionnaire | Sleep hours - weekends | SLD013 |
| Sleep | OSA | Categorical | SLQ | Questionnaire | How often do you snore? | SLQ030 |
| Sleep | OSA | Categorical | SLQ | Questionnaire | How often do you snort or stop breathing? | SLQ040 |
| Sleep | OSA | Categorical | SLQ | Questionnaire | How often feel overly sleepy during day? | SLQ120 |
| Activity | Sedentary behavior (continuous) | Continuous | PAQ | Questionnaire | Minutes sedentary activity | PAQ680 |
| Activity | Sedentary behavior (categorical) | Categorical | PAQ | Questionnaire | Minutes sedentary activity (<4h, 4-8h, ≥8h) | PAQ680 |
| Activity | BMI | Continuous | BMX | Examination | Body Mass Index | BMXBMI |
| Activity | BMI group | Categorical | BMX | Examination | BMI categories (Normal<25, Overweight 25-30, Obesity≥30) | BMXBMI |
| MetS | MetS | Categorical (Yes/No) | Multiple | Multiple | ≥3 of the following conditions: |  |
| MetS | 1.Central obesity | Categorical | BMX | Examination | Waist circumference >102 cm (M), >88 cm (F) | BMIWAIST |
| MetS | 2.Hyperglycemia | Categorical | GLU | Laboratory | Fasting Glucose >5.6 mmol/L or diabetes history | LBXGLU |
| MetS | 2. Hyperglycemia | Categorical | BIOPRO | Laboratory | Glucose, refrigerated serum | LBDSGLSI |
| MetS | 2. Hyperglycemia | Categorical | DIQ | Questionnaire | Doctor told you have diabetes | DIQ010 |
| MetS | 2. Hyperglycemia | Categorical | DSQIDS | Dietary | To maintain blood sugar, diabetes | DSD128T |
| MetS | 3. Hypertension | Categorical | BPQ | Questionnaire | Ever told you had high blood pressure | BPQ020 |
| MetS | 3. Hypertension | Categorical | BPQ | Questionnaire | Taking prescription for hypertension | BPQ040A |
| MetS | 4.High Triglycerides | Categorical | BIOPRO | Laboratory | Triglycerides ≥1.70 mmol/L | LBDSTRSI |
| MetS | 5. Low HDL-C | Categorical | HDL | Laboratory | HDL-C <1.04 mmol/L (M), <1.3 mmol/L (F) | LBDHDDSI |
| MetS | 5. Low HDL-C | Categorical | BPQ | Questionnaire | Told to take prescription for cholesterol | BPQ090D |

**Note:** *MetS:* Metabolic Syndrome; *OSA:* Obstructive sleep apnea.
