# Supplementary material for: Association between weekend catch-up sleep and metabolic syndrome: A cross-sectional study
Source: Medicine (Baltimore). 2026 Jun 26;105(26):e49299. doi: 10.1097/MD.0000000000049299 (PMC13313639; doi:10.1097/MD.0000000000049299)
Supplement: Supplementary file 2 [file medi-105-e49299-s002.doc]

**Table S2. Participant Characteristics WCS group (n = 7,658)**

| Variable | Total | Decreased | No change | Short | Moderate | Long | P value |
| --- | --- | --- | --- | --- | --- | --- | --- |
| MetS |  |  |  |  |  |  | 0.01 |
| No | 5574(75.70) | 911(74.96) | 1272(75.52) | 331(76.24) | 1933(73.32) | 1127(80.19) |  |
| Yes | 2084(24.30) | 323(25.04) | 442(24.48) | 105(23.76) | 852(26.68) | 362(19.81) |  |
| Age | 47.00(0.50) | 44.33(0.63) | 41.85(0.60) | 43.52(1.07) | 52.95(0.59) | 45.04(0.59) | < 0.0001 |
| Age group |  |  |  |  |  |  | < 0.0001 |
| 20-44 | 3118(45.71) | 588(52.61) | 899(57.78) | 210(54.59) | 743(31.88) | 678(49.51) |  |
| 45-64 | 3002(36.92) | 447(33.31) | 677(37.93) | 193(38.66) | 1086(36.19) | 599(39.18) |  |
| >=65 | 1538(17.37) | 199(14.08) | 138( 4.29) | 33( 6.75) | 956(31.93) | 212(11.32) |  |
| sex |  |  |  |  |  |  | 0.13 |
| Female | 3926(51.08) | 625(50.06) | 886(48.59) | 244(51.21) | 1373(51.00) | 798(54.33) |  |
| Male | 3732(48.92) | 609(49.94) | 828(51.41) | 192(48.79) | 1412(49.00) | 691(45.67) |  |
| Race |  |  |  |  |  |  | < 0.0001 |
| Mexican American | 2458(62.26) | 367(59.74) | 390(51.82) | 147(64.49) | 1061(67.29) | 493(65.15) |  |
| Non-Hispanic Black | 2094(11.35) | 439(16.02) | 533(14.24) | 99( 8.03) | 632( 9.14) | 391( 9.94) |  |
| Non-Hispanic White | 934( 8.52) | 124( 8.01) | 301(13.10) | 59( 9.87) | 280( 6.46) | 170( 7.44) |  |
| Other | 2172(17.87) | 304(16.23) | 490(20.85) | 131(17.60) | 812(17.11) | 435(17.47) |  |
| Education |  |  |  |  |  |  | 0.002 |
| Below high school | 566( 3.47) | 71(2.94) | 159(5.14) | 22(2.61) | 227(3.52) | 87(2.41) |  |
| High school education or more | 7087(96.50) | 1163(97.06) | 1554(94.86) | 414(97.39) | 2555(96.48) | 1401(97.59) |  |
| Marital |  |  |  |  |  |  | 0.01 |
| marry | 6086(80.06) | 934(76.79) | 1296(77.13) | 363(83.77) | 2313(82.81) | 1180(79.51) |  |
| Not married | 1572(19.94) | 300(23.21) | 418(22.87) | 73(16.23) | 472(17.19) | 309(20.49) |  |
| BMI | 29.83(0.20) | 30.11(0.28) | 30.51(0.30) | 30.18(0.53) | 29.34(0.24) | 29.67(0.37) | < 0.001 |
| BMI |  |  |  |  |  |  | 0.05 |
| Normal | 1927(26.41) | 312(26.11) | 408(26.38) | 105(25.37) | 713(27.21) | 389(25.61) |  |
| Overweight | 2410(31.37) | 377(32.03) | 525(27.48) | 134(26.29) | 919(33.30) | 455(33.02) |  |
| Obesity | 3321(42.22) | 545(41.86) | 781(46.15) | 197(48.34) | 1153(39.49) | 645(41.37) |  |
| Social jetlag |  |  |  |  |  |  | < 0.0001 |
| <2h | 5965(79.27) | 1026(84.86) | 785(46.70) | 341(78.37) | 2600(93.93) | 1213(84.65) |  |
| >=2h | 1620(20.15) | 198(15.14) | 892(53.30) | 94(21.63) | 179(6.07) | 257(15.35) |  |
| Smoke |  |  |  |  |  |  | 0.002 |
| Never | 1704(25.06) | 239(22.18) | 313(23.29) | 84(22.82) | 745(27.95) | 323(24.67) |  |
| Former | 4499(57.59) | 685(55.36) | 1094(59.45) | 299(67.40) | 1505(53.95) | 916(60.46) |  |
| Now | 1455(17.35) | 310(22.46) | 307(17.27) | 53( 9.78) | 535(18.11) | 250(14.87) |  |
| Alcohol |  |  |  |  |  |  | < 0.0001 |
| Mild | 1656(26.12) | 296(30.87) | 441(32.75) | 91(23.47) | 500(20.14) | 328(27.09) |  |
| Moderate | 4727(55.36) | 707(50.07) | 982(49.16) | 250(49.08) | 1890(63.81) | 898(52.90) |  |
| Heavy | 1275(18.53) | 231(19.06) | 291(18.09) | 95(27.45) | 395(16.05) | 263(20.01) |  |
| weekday duration, h |  |  |  |  |  |  | < 0.0001 |
| <=6 | 1542(17.18) | 91( 4.86) | 698(39.11) | 62(12.62) | 443(13.65) | 248(11.75) |  |
| >=9 | 1627(18.60) | 495(38.72) | 121( 5.17) | 33( 5.93) | 751(24.36) | 227(11.76) |  |
| 6-9 | 4489(64.22) | 648(56.42) | 895(55.72) | 341(81.46) | 1591(61.99) | 1014(76.49) |  |
| Sedentary behavior, n (%) |  |  |  |  |  |  | < 0.001 |
| <4 hours | 1325(20.69) | 202(20.36) | 289(20.74) | 90(25.93) | 441(17.80) | 303(24.59) |  |
| 4-8 hours | 2481(27.62) | 410(31.46) | 647(35.20) | 132(24.28) | 868(25.47) | 424(22.80) |  |
| >=8 hours | 3805(51.18) | 615(48.18) | 770(44.07) | 210(49.79) | 1454(56.73) | 756(52.62) |  |
| OSA |  |  |  |  |  |  | 0.1 |
| No | 3789(50.65) | 651(53.46) | 822(49.98) | 228(49.57) | 1323(47.94) | 765(54.17) |  |
| Yes | 3869(49.35) | 583(46.54) | 892(50.02) | 208(50.43) | 1462(52.06) | 724(45.83) |  |
| SBP, mm Hg | 121.33(0.36) | 120.58(0.55) | 120.22(0.52) | 119.21(1.26) | 123.79(0.63) | 119.51(0.66) | < 0.001 |
| DBP, mm Hg | 74.21(0.27) | 74.15(0.46) | 75.14(0.48) | 74.37(0.95) | 73.91(0.26) | 73.83(0.55) | 0.24 |
| TGs, mmol/L | 1.25(0.03) | 1.19(0.06) | 1.25(0.06) | 1.19(0.07) | 1.32(0.04) | 1.21(0.06) | 0.4 |
| HDL, mmol/l | 1.38(0.01) | 1.35(0.01) | 1.34(0.02) | 1.39(0.02) | 1.41(0.02) | 1.41(0.02) | 0.002 |
| WC, cm | 100.52(0.52) | 100.20(0.74) | 101.19(0.59) | 101.37(1.32) | 100.66(0.61) | 99.62(0.92) | 0.24 |
| FG, mmol/l | 6.06(0.05) | 5.96(0.09) | 6.12(0.13) | 5.86(0.11) | 6.17(0.08) | 5.95(0.07) | 0.25 |

Values were numbers (weighted percentages), and survey-designed Chi-square tests calculated the P value.

**Note:** BMI Body Mass Index, WCS Weekend Catch-Up Sleep, MetS Metabolic Syndrome; WC Waist circumference, OSA obstructive sleep apnea; SBP Systolic blood pressure, DBP Diastolic blood pressure, FG Fasting glucose, TGs Triglycerides, HDL-C High-density lipoprotein cholesterol.
