# Supplementary material for: Association between weekend catch-up sleep and metabolic syndrome: A cross-sectional study
Source: Medicine (Baltimore). 2026 Jun 26;105(26):e49299. doi: 10.1097/MD.0000000000049299 (PMC13313639; doi:10.1097/MD.0000000000049299)
Supplement: Supplementary file 3 [file medi-105-e49299-s003.doc]

**Table S3 Univariate analysis of the association between MetS and covariates.**

| **Variable** | **OR (95 % CI)** | **P value** |
| --- | --- | --- |
| **Age** | 0.007(0.006,0.008) | <0.001 |
| Age group |  |  |
| 20-44 | ref | ref |
| 45-64 | 2.920(2.439,3.495) | <0.001 |
| >=65 | 4.401(3.350,5.782) | <0.001 |
| **Gender** |  |  |
| Female (ref) | ref | ref |
| Male | 0.893(0.740,1.078) | 0.226 |
| **Race** |  |  |
| Non-Hispanic White | ref | ref |
| Non-Hispanic Black | 0.934(0.719,1.214) | 0.596 |
| Mexican American | 1.019(0.779,1.332) | 0.887 |
| Other | 0.891(0.713,1.114) | 0.296 |
| **Educational Level** |  |  |
| Less than high school | ref | ref |
| More than high school | 0.603(0.466,0.781) | <0.001 |
| **Marital status** |  |  |
| Married/living with partner | ref | ref |
| Separated/divorced/widowed/never married | 0.475(0.383,0.589) | <0.001 |
| **Sedentary behavior** |  |  |
| <4 hours | ref |  |
| 4-8 hours | 1.281(1.052,1.560) | 0.016 |
| >=8 hours | 1.411(1.062,1.873) | 0.020 |
| **Smoking** |  |  |
| Never smoker Current smoker | ref | ref |
| Former smoker | 0.601(0.456,0.793) | <0.001 |
| Current smoker | 0.710(0.568,0.887) | 0.004 |
| **Alcohol Drinking** |  |  |
| Mild | ref | ref |
| Moderate | 1.729(1.420,2.104) | <0.001 |
| Heavy | 1.059(0.769,1.458) | 0.716 |
| weekend catch-up sleep time (hours) | -0.002(-0.009,0.005) | 0.546 |
| **Weekday sleep duration** |  |  |
| 6-9 | ref | ref |
| <=6 | 1.235(0.955,1.597) | 0.103 |
| >=9 | 1.175(0.931,1.482) | 0.165 |
| **OSA** |  |  |
| No | ref | ref |
| Yes | 1.951(1.720,2.214) | <0.001 |
| **Social jetlag** |  |  |
| No | ref | ref |
| Yes | 0.699(0.566,0.864) | 0.002 |

**Note:** MetS, Metabolic syndrome; OR, odds ratio; OSA: Obstructive sleep apnea.
