# Supplementary material for: Association between weekend catch-up sleep and metabolic syndrome: A cross-sectional study
Source: Medicine (Baltimore). 2026 Jun 26;105(26):e49299. doi: 10.1097/MD.0000000000049299 (PMC13313639; doi:10.1097/MD.0000000000049299)
Supplement: Supplementary file 4 [file medi-105-e49299-s004.doc]

| **Table S4. Marginal odds ratios (ORs) with 95% CIs for associations between weekend catch-up sleep (WCS) and metabolic syndrome components.** | | | | | | | | | | | | | | |
| --- | --- | --- | --- | --- | --- | --- | --- | --- | --- | --- | --- | --- | --- | --- |
|  |  |  |  |  |  |  |  |  |  |  |  |  |  |  |
|  | Hyperglycemia |  |  | Lower HDL-C |  |  | Higher TGs |  |  | Obesity |  |  | Hypertension |  |
|  | OR (95% CI) | P value |  | OR (95% CI) | P value |  | OR (95% CI) | P value |  | OR (95% CI) | P value |  | OR (95% CI) | P value |
| WCS (duration > 0 h) |  |  |  |  |  |  |  |  |  |  |  |  |  |  |
| No | ref |  |  | ref |  |  | ref |  |  | ref |  |  | ref |  |
| Yes | 1.1(0.97,1.24) | 0.153 |  | 1.09(0.93,1.29) | 0.281 |  | 1(0.79,1.26) | 0.998 |  | 1.13(0.93,1.37) | 0.205 |  | 1.01(0.85,1.2) | 0.899 |
| WCS duration (continuous) | 0.044(-0.014,0.102) | 0.119 |  | 0.039(-0.015,0.093) | 0.138 |  | 0.019(-0.071,0.109) | 0.646 |  | 0.043(-0.028,0.113) | 0.204 |  | 0.041(-0.019,0.101) | 0.154 |
| Decreased WCS | ref |  |  | ref |  |  | ref |  |  | ref |  |  | ref |  |
| No change | 1.17(0.86,1.59) | 0.524 |  | 1.13(0.89,1.43) | 0.533 |  | 1.22(0.72,2.04) | 0.73 |  | 1.11(0.73,1.67) | 0.892 |  | 0.94(0.65,1.36) | 0.962 |
| Short WCS | 1.12(0.73,1.71) | 0.882 |  | 0.99(0.6,1.64) | 1 |  | 0.79(0.37,1.69) | 0.818 |  | 1.15(0.71,1.84) | 0.851 |  | 0.84(0.6,1.18) | 0.531 |
| Moderate WCS | 0.98(0.73,1.32) | 0.996 |  | 0.84(0.66,1.09) | 0.292 |  | 1.06(0.64,1.76) | 0.98 |  | 0.8(0.56,1.15) | 0.356 |  | 0.64(0.46,0.89) | 0.003 |
| Long WCS | 1.02(0.78,1.32) | 0.996 |  | 0.87(0.63,1.21) | 0.673 |  | 1.01(0.61,1.65) | 1 |  | 0.85(0.56,1.28) | 0.699 |  | 0.6(0.43,0.83) | <0.001 |
| *P* for trend |  | 0.54 |  |  | 0.062 |  |  | 0.792 |  |  | 0.062 |  |  | **0.001** |

Notes:

Data were adjusted for age, sex, race, educational level, living with partner status, smoking status, alcohol drinking status, sedentary behavior, and social jetlag and OSA.

Abbreviations: WCS, weekend catch-up sleep; OR, odds ratio; CI, confidence interval; OSA, obstructive sleep apnea.
