# Supplementary material for: Association between weekend catch-up sleep and metabolic syndrome: A cross-sectional study
Source: Medicine (Baltimore). 2026 Jun 26;105(26):e49299. doi: 10.1097/MD.0000000000049299 (PMC13313639; doi:10.1097/MD.0000000000049299)
Supplement: Supplementary file 5 [file medi-105-e49299-s005.doc]

**Table S5.** **Subgroup Analysis of the Association between Weekend Catch-up Sleep (WCS) Duration and MetS**

| Character | Decreased | No change | p | Short | p | Moderate | p | Long | p | p for interaction |
| --- | --- | --- | --- | --- | --- | --- | --- | --- | --- | --- |
| Weekday sleep duration (h) |  |  |  |  |  |  |  |  |  |  |
| 6-9 | ref | 1.16 (0.73, 1.84) | 0.8 | 0.91 (0.46, 1.82) | 0.98 | **0.6 (0.39, 0.92)** | **0.01** | **0.63 (0.41, 0.97)** | **0.03** | 0.139 |
| >=9 | ref | 0.84 (0.37, 1.88) | 0.92 | 2.63 (0.44, 15.81) | 0.48 | 0.91 (0.57, 1.45) | 0.93 | 0.95 (0.48, 1.86) | 0.99 |  |
| <=6 | ref | 1.8 (0.72, 4.5) | 0.32 | 1.28 (0.28, 5.94) | 0.96 | 1.36 (0.47, 3.94) | 0.85 | 1.4 (0.54, 3.62) | 0.76 |  |
| Age group |  |  |  |  |  |  |  |  |  |  |
| 20-44 | ref | 1.26 (0.65, 2.45) | 0.76 | 0.74 (0.26, 2.07) | 0.84 | 0.89 (0.55, 1.44) | 0.89 | 0.81 (0.5, 1.31) | 0.63 | 0.465 |
| 45-64 | ref | 1.05 (0.49, 2.26) | 1 | 1.25 (0.67, 2.33) | 0.76 | 0.77 (0.42, 1.44) | 0.67 | 0.63 (0.38, 1.06) | 0.1 |  |
| >=65 | ref | 1.16 (0.43, 3.09) | 0.97 | 1.44 (0.64, 3.24) | 0.62 | 0.63 (0.33, 1.22) | 0.26 | 0.81 (0.4, 1.64) | 0.83 |  |
| Sex |  |  |  |  |  |  |  |  |  |  |
| Female | ref | 1.29 (0.92, 1.81) | 0.2 | 1.15 (0.59, 2.25) | 0.92 | 0.85 (0.52, 1.38) | 0.78 | 0.81 (0.45, 1.43) | 0.73 | 0.88 |
| Male | ref | 1.07 (0.56, 2.04) | 0.99 | 0.88 (0.39, 2.02) | 0.97 | **0.62 (0.43, 0.91)** | **0.01** | 0.65 (0.39, 1.1) | 0.14 |  |
| Race |  |  |  |  |  |  |  |  |  |  |
| Non-Hispanic White | ref | 1.11 (0.44, 2.81) | 0.98 | 0.79 (0.16, 3.93) | 0.97 | 0.78 (0.36, 1.69) | 0.8 | 0.78 (0.35, 1.77) | 0.83 | 0.006 |
| Non-Hispanic Black | ref | 1.11 (0.69, 1.77) | 0.92 | 0.98 (0.57, 1.68) | 1 | 0.96 (0.6, 1.53) | 0.99 | 1.14 (0.74, 1.74) | 0.83 |  |
| Mexican American | ref | 1.19 (0.66, 2.14) | 0.84 | 1.04 (0.49, 2.19) | 1 | 0.63 (0.39, 1.03) | 0.07 | **0.59 (0.38, 0.92)** | **0.01** |  |
| Other | ref | 1.13 (0.49, 2.6) | 0.97 | 0.98 (0.43, 2.2) | 1 | 0.84 (0.43, 1.65) | 0.88 | 0.85 (0.44, 1.63) | 0.89 |  |
| Sedentary behavior, hours |  |  |  |  |  |  |  |  |  |  |
| <4 | ref | 0.9 (0.46, 1.79) | 0.97 | 0.65 (0.25, 1.71) | 0.63 | 0.86 (0.54, 1.39) | 0.82 | 0.67 (0.41, 1.09) | 0.15 | 0.056 |
| 4-8 | ref | 1.4 (0.82, 2.39) | 0.35 | 1.7 (0.9, 3.23) | 0.14 | 0.72 (0.45, 1.16) | 0.27 | 0.99 (0.62, 1.57) | 1 |  |
| >=8 | ref | 1.15 (0.56, 2.35) | 0.94 | 0.54 (0.17, 1.64) | 0.45 | 0.64 (0.4, 1.03) | 0.07 | 0.38 (0.17, 0.84) | 0.01 |  |
| alcohol |  |  |  |  |  |  |  |  |  |  |
| Mild | ref | 0.99 (0.48, 2.06) | 1 | 0.36 (0.1, 1.25) | 0.14 | 0.59 (0.32, 1.09) | 0.12 | **0.34 (0.17, 0.67)** | 0 | 0.189 |
| Moderate | ref | 1.16 (0.63, 2.12) | 0.9 | 1.21 (0.64, 2.27) | 0.84 | 0.79 (0.52, 1.21) | 0.46 | 0.83 (0.51, 1.35) | 0.72 |  |
| Heavy | ref | 1.42 (0.72, 2.84) | 0.52 | 1.83 (0.64, 5.24) | 0.42 | 0.75 (0.46, 1.23) | 0.42 | 1.08 (0.52, 2.25) | 0.98 |  |
| OSA |  |  |  |  |  |  |  |  |  |  |
| No | ref | 1.24 (0.72, 2.12) | 0.7 | 0.92 (0.38, 2.27) | 0.99 | 0.66 (0.43, 1.01) | 0.06 | 0.62 (0.38, 1) | 0.05 | 0.372 |
| Yes | ref | 1.12 (0.63, 1.99) | 0.94 | 1.12 (0.57, 2.22) | 0.95 | 0.78 (0.52, 1.18) | 0.39 | 0.81 (0.57, 1.17) | 0.43 |  |
| Social jetlag |  |  |  |  |  |  |  |  |  |  |
| No | ref | 1.37 (0.94, 1.99) | 0.13 | 1.16 (0.67, 2.03) | 0.86 | 0.8 (0.6, 1.06) | 0.16 | 0.72 (0.48, 1.08) | 0.14 | 0.843 |
| Yes | ref | 0.64 (0.29, 1.45) | 0.47 | 0.51 (0.13, 2.01) | 0.55 | **0.34 (0.14, 0.85)** | 0.01 | 0.72 (0.37, 1.41) | 0.55 |  |

Footnotes:

Data were adjusted for sex, age, race, educational level, marital status, smoking status, alcohol drinking status, social jetlag, and OSA.

Abbreviations: WCS, weekend catch-up sleep; OR, odds ratio; CI, confidence interval;

Reference group: Decreased WCS.

In the table, bold type indicates a P value < 0.05.
