# Supplementary material for: Association between weekend catch-up sleep and metabolic syndrome: A cross-sectional study
Source: Medicine (Baltimore). 2026 Jun 26;105(26):e49299. doi: 10.1097/MD.0000000000049299 (PMC13313639; doi:10.1097/MD.0000000000049299)
Supplement: Supplementary file 6 [file medi-105-e49299-s006.doc]

**Table S6. Participant Characteristics by Race/Ethnicity in the NHANES 2017–2020 Cycles (n = 7,658)**

| Variable | Total | Mexican American | Non-Hispanic Black | Non-Hispanic White | Other | Pvalue |
| --- | --- | --- | --- | --- | --- | --- |
| Age | 47.00(0.50) | 48.86(0.65) | 45.04(0.45) | 40.79(0.75) | 44.76(0.64) | < 0.001 |
| Age group |  |  |  |  |  | < 0.001 |
| >=65 | 1538(17.37) | 651(20.86) | 416(13.38) | 120( 6.72) | 351(12.80) |  |
| 20-44 | 3118(45.71) | 920(40.58) | 808(50.51) | 448(62.60) | 942(52.50) |  |
| 45-64 | 3002(36.92) | 887(38.56) | 870(36.12) | 366(30.68) | 879(34.71) |  |
| Education |  |  |  |  |  | < 0.001 |
| Below high school | 566( 3.47) | 40( 0.82) | 41( 1.59) | 242(15.63) | 243( 8.13) |  |
| High school or more | 7087(96.50) | 2418(99.18) | 2053(98.41) | 690(84.37) | 1926(91.87) |  |
| Marital |  |  |  |  |  | < 0.001 |
| Marry | 6086(80.06) | 2073(83.13) | 1450(65.85) | 778(78.94) | 1785(78.92) |  |
| Not married | 1572(19.94) | 385(16.87) | 644(34.15) | 156(21.06) | 387(21.08) |  |
| BMI | 29.83(0.20) | 29.79(0.27) | 31.25(0.19) | 30.68(0.26) | 28.67(0.25) | < 0.001 |
| BMI group |  |  |  |  |  | < 0.001 |
| Normal | 1927(26.41) | 631(27.55) | 461(23.37) | 144(16.03) | 691(29.30) |  |
| Overweight | 2410(31.37) | 737(30.43) | 552(26.84) | 324(34.14) | 797(36.23) |  |
| Obesity | 3321(42.22) | 1090(42.03) | 1081(49.79) | 466(49.83) | 684(34.47) |  |
| WCS | 0.69(0.03) | 0.59(0.03) | 0.78(0.04) | 1.07(0.07) | 0.82(0.05) | < 0.001 |
| WCS group |  |  |  |  |  | < 0.001 |
| Decreased sleep duration | 1234(15.12) | 367(14.51) | 439(21.35) | 124(14.22) | 304(13.73) |  |
| No change in sleep duration | 1714(20.95) | 390(17.43) | 533(26.28) | 301(32.22) | 490(24.44) |  |
| Short catch-up sleep duration | 436( 6.51) | 147(6.74) | 99(4.61) | 59(7.54) | 131(6.41) |  |
| Moderate catch-up sleep duration | 2785(35.90) | 1061(38.80) | 632(28.91) | 280(27.22) | 812(34.38) |  |
| Long catch-up sleep duration | 1489(21.52) | 493(22.52) | 391(18.85) | 170(18.80) | 435(21.04) |  |
| Social jetlag group |  |  |  |  |  | < 0.001 |
| No | 5965(79.27) | 2080(83.39) | 1461(69.44) | 695(72.33) | 1729(76.94) |  |
| Yes | 1620(20.15) | 364(16.61) | 591(30.56) | 236(27.67) | 429(23.06) |  |
| Smoke |  |  |  |  |  | < 0.001 |
| Former | 4499(57.59) | 1188(53.18) | 1226(63.70) | 604(66.25) | 1481(64.94) |  |
| Never | 1704(25.06) | 728(29.08) | 369(14.73) | 215(20.92) | 392(19.57) |  |
| Now | 1455(17.35) | 542(17.74) | 499(21.57) | 115(12.83) | 299(15.50) |  |
| Alcohol |  |  |  |  |  | < 0.001 |
| Mild | 1656(26.12) | 603(27.59) | 394(19.95) | 294(35.52) | 365(20.43) |  |
| Moderate | 4727(55.36) | 1398(53.05) | 1323(60.65) | 487(46.51) | 1519(64.23) |  |
| Heavy | 1275(18.53) | 457(19.36) | 377(19.39) | 153(17.97) | 288(15.34) |  |
| PIR |  |  |  |  |  | < 0.001 |
| <1 | 1278(11.21) | 302( 8.15) | 459(23.04) | 187(23.00) | 330(17.74) |  |
| 1-3 | 2789(29.87) | 907(29.14) | 787(43.37) | 366(46.21) | 729(37.92) |  |
| >=3 | 2554(47.97) | 1042(62.71) | 529(33.60) | 216(30.79) | 767(44.34) |  |
| Weekday duration |  |  |  |  |  | < 0.001 |
| <=6 | 1542(17.18) | 381(14.57) | 587(27.09) | 162(17.66) | 412(19.74) |  |
| >=9 | 1627(18.60) | 516(17.50) | 464(22.37) | 207(20.09) | 440(19.35) |  |
| 6-9 | 4489(64.22) | 1561(67.94) | 1043(50.54) | 565(62.25) | 1320(60.91) |  |
| Sedentary behavior, n (%) |  |  |  |  |  | < 0.0001 |
| <4 hours | 2481(27.62) | 608(22.94) | 621(28.82) | 461(46.44) | 791(34.96) |  |
| 4-8 hours | 3805(51.18) | 1311(53.66) | 1090(53.15) | 377(42.54) | 1027(46.90) |  |
| >=8 hours | 1325(20.69) | 524(23.40) | 367(18.03) | 91(11.02) | 343(18.14) |  |

Values were numbers (weighted percentages), and survey-designed Chi-square tests calculated the *P* value.

**Note:** *BMI* Body Mass Index, *WCS* Weekend Catch-Up Sleep, *MetS* Metabolic Syndrome,PIR .
