# Supplementary material for: Association between weekend catch-up sleep and metabolic syndrome: A cross-sectional study
Source: Medicine (Baltimore). 2026 Jun 26;105(26):e49299. doi: 10.1097/MD.0000000000049299 (PMC13313639; doi:10.1097/MD.0000000000049299)
Supplement: Supplementary file 7 [file medi-105-e49299-s007.doc]

**Table S7. Sensitivity analysis of associations between weekday sleep duration (3–12 hours) and metabolic syndrome components using logistic regression: Weekend catch-up sleep (WCS) categories**

| Variables | MetS | |  | Hyperglycemia | |  | Lower HDL | |  | Higher TG | |  | Obesity | |  | Hypertension | |
| --- | --- | --- | --- | --- | --- | --- | --- | --- | --- | --- | --- | --- | --- | --- | --- | --- | --- |
|  | OR (95% CI) | P value |  | OR (95% CI) | P value |  | OR (95% CI) | P value |  | OR (95% CI) | P value |  | OR (95% CI) | P value |  | OR (95% CI) | P value |
| Decreased WCS | ref |  |  | ref |  |  | ref |  |  | ref |  |  | ref | ref |  | ref |  |
| No change | 1.11(0.76,1.61) | 0.865 |  | 1.17(0.86,1.59) | 0.53 |  | 1.12(0.88,1.42) | 0.587 |  | 1.21(0.72,2.05) | 0.738 |  | 1.11(0.73,1.68) | 0.893 |  | 0.94(0.65,1.36) | 0.954 |
| Short WCS | 1(0.58,1.71) | 1 |  | 1.12(0.73,1.71) | 0.884 |  | 0.99(0.6,1.63) | 0.999 |  | 0.79(0.37,1.69) | 0.814 |  | 1.15(0.71,1.84) | 0.848 |  | 0.84(0.6,1.17) | 0.498 |
| Moderate WCS | 0.7(0.51,0.95) | 0.018 |  | 0.98(0.73,1.33) | 0.997 |  | 0.84(0.65,1.08) | 0.258 |  | 1.06(0.64,1.76) | 0.981 |  | 0.8(0.56,1.16) | 0.384 |  | **0.63(0.45,0.88)** | **0.003** |
| Long WCS | 0.69(0.5,0.95) | 0.018 |  | 1.01(0.78,1.33) | 0.997 |  | 0.86(0.62,1.2) | 0.632 |  | 1(0.61,1.65) | 1 |  | 0.85(0.56,1.28) | 0.703 |  | **0.6(0.43,0.82)** | **<0.001** |

Footnotes:

Data were adjusted for age, sex, race, educational level, living with partner status, smoking status, alcohol drinking status, sedentary behavior, and social jetlag and OSA.

Abbreviations: WCS, weekend catch-up sleep; OR, odds ratio; CI, confidence interval;

Reference group: Decreased WCS.

*P < 0.05.
